# Characterization of Fine Particulate Matter and Associated Health Burden in Nanjing

**DOI:** 10.3390/ijerph15040602

**Published:** 2018-03-27

**Authors:** Dongyang Nie, Mindong Chen, Yun Wu, Xinlei Ge, Jianlin Hu, Kai Zhang, Pengxiang Ge

**Affiliations:** 1 Jiangsu Key Laboratory of Atmospheric Environment Monitoring and Pollution Control, Collaborative Innovation Center of Atmospheric Environment and Equipment Technology, School of Environmental Science and Engineering, Nanjing University of Information Science & Technology, Nanjing 210044, China; dynie@nuist.edu.cn (D.N.); caxinra@163.com (X.G.); hu_jianlin@126.com (J.H.); zhangkai@nuist.edu.cn (K.Z.); gpx0904@126.com (P.G.); 2 School of Atmospheric Physics, Nanjing University of Information Science & Technology, Nanjing 210044, China

**Keywords:** fine particulate matter, health burden, integrated exposure-response model, Nanjing

## Abstract

Particulate matter (PM) air pollution has become a serious environmental problem in Nanjing and poses great health risks to local residents. In this study, characteristics of particulate matter with an aerodynamic diameter less than 2.5 μm (PM_2.5_) over Nanjing were analyzed using hourly and daily averaged PM_2.5_ concentrations and meteorological parameters collected from nine national monitoring sites during the period of March 2014 to February 2017. Then, the integrated exposure-response (IER) model was applied to assess premature mortality, years of life lost (YLL) attributable to PM_2.5_, and mortality benefits due to PM_2.5_ reductions. The concentrations of PM_2.5_ varied among hours, seasons and years, which can be explained by differences in emission sources, secondary formations and meteorological conditions. The decreased ratio of PM_2.5_ to CO suggested that secondary contributions decreased while the relative contributions of vehicle exhaust increased from increased CO data. According to the values of attributable fractions (AF), stroke was the major cause of death, followed by ischemic heart disease (IHD), lung cancer (LC) and chronic obstructive pulmonary disease (COPD). The estimated total deaths in Nanjing due to PM_2.5_ were 12,055 and 10,771, leading to 98,802 and 87,647 years of life lost in 2014 and 2015, respectively. The elderly and males had higher health risks than youngsters and females. When the PM_2.5_ concentrations meet the World Health Organization (WHO) Air Quality Guidelines (AQG) of 10 μg/m^3^, 84% of the premature deaths would be avoided, indicating that the Nanjing government needs to adopt more stringent measure to reduce PM pollution and enhance the health benefits.

## 1. Introduction

Along with rapid industrialization and urbanization, China has been experiencing severe air pollution problems, especially in particulate matter (PM) pollution. The annual average concentrations of particles with an aerodynamic diameter equal to or less than 2.5 μm (PM_2.5_) in most Chinese cities have greatly exceeded the World Health Organization (WHO) guideline value of 10 μg/m^3^ [1,2,3,4,5,6,7]. The Yangtze River Delta (YRD) is one of the most developed and polluted regions in China, and it has been identified as a key area for the Air Pollution Prevention and Control Action Plan since 2013 [8]. Nanjing, located in the middle and lower reaches of the Yangtze River region, is the capital city of Jiangsu province, with an area of 6596 km^2^ and a population of 8.27 million by the end of 2016. Due to the well-developed industry and increasing number of vehicles [9], severe PM_2.5_ pollution was seen in Nanjing from historical data. Considering its high population density, adverse impacts on human health could be expected, or, in fact, observed [10,11].

PM_2.5_ have been proven to be the triggers for a variety of illnesses, such as bronchitis, asthma, diabetes, chronic obstructive pulmonary disease (COPD), and cardiovascular disease [12,13,14,15,16,17,18,19]. The International Agency for Research on Cancer (IARC) has classified outdoor air pollution and air pollution-derived PM as carcinogenic to humans. The integrated exposure–response (IER) function was developed for the Global Burden of Disease Study by integrating PM_2.5_ information and producing a more reasonable prediction of the relative risks (RRs) [20]. It has been employed in a number of recent mortality estimates attributed to PM_2.5_ exposure [20,21,22,23,24,25]. To date, long-term exposure to PM_2.5_ has been considered to induce premature mortality from COPD, stroke, ischemic heart disease (IHD), lung cancer (LC) and acute lower respiratory infection (ALRI) [21,22,23,24,26,27,28,29,30,31]. Premature mortality due to air pollution in China have been widely reported in previous studies. Lelieveld et al. [32] estimated that outdoor air pollution in China caused about 1.36 million premature deaths in 2010. Liu et al. [27] reported that 1.37 million adult premature mortalities in China were related with PM_2.5_ in 2013. Cohen et al. [28] estimated that PM_2.5_ caused 1.1 million premature deaths by a 25-year trends of the global burden of disease study over China in 2015.

To provide a better understanding of the effects of implementing the toughest ever air pollution control policy, premature mortality and years of life lost (YLL) serves two important health-related indicators to be tested. Zhang et al. [33] has estimated the improvements in air quality and the corresponding health benefits since the beginning of the Clean Air Action Plan in China. Song et al. [21] reported that the mortality benefits would be 24.0%, 44.8%, 70.8%, and 85.2% of the total current mortalities (1.5 million) when the population-weighted average (PWA) PM_2.5_ concentrations in China meets the World Health Organization (WHO) Air Quality Guidelines (AQG: 10 μg/m^3^) and three interim targets (ITs, IT-1: 35 μg/m^3^, IT-2: 25 μg/m^3^, IT-3: 15 μg/m^3^), respectively. Hu et al. [22] also pointed out that a 30% excess mortality reduction in China required a 50% reduction in PM_2.5_ throughout the country, and the necessary reduction for the Beijing–Tianjin–Hebei, Jiangsu–Zhejiang–Shanghai, and Pearl River Delta regions were 62%, 50%, and 38%, respectively. Those previous studies paint general pictures of PM_2.5_-induced mortality over China and large variations in different regions due to different emission sources and climate changes. However, little attention has been paid to the health burdens and benefits at the city level, e.g., Nanjing.

Therefore, the objective of this study is to investigate the characteristics of PM_2.5_, the related health burden and its responses to different PM_2.5_ reduction targets at the city level in China. Nanjing has a typical subtropical monsoon climate and four distinct seasons. In this study, we investigated the temporal variation of PM_2.5_ in Nanjing, as well as the influences of meteorological parameters on PM_2.5_. Hourly and daily averaged PM_2.5_ concentrations were collected from nine national monitoring sites during the period March 2014 to February 2017. Then, we calculated the cause-specific and the age-specific premature mortality and YLL attributable to PM_2.5_ exposure based on the IER model. Finally, we estimated the potentially avoidable premature mortality in situations in which ambient PM_2.5_ concentrations in Nanjing are reduced to four different standard levels (AQG and three WHO ITs).

## 2. Materials and Methods

### 2.1. Data Collection: Meteorological Conditions, PM_2.5_ and Other Air Pollutants

Mass concentrations of PM_2.5_ and other air pollutants used in this study were downloaded from the website of the Chinese Environmental Protection Bureau (http://www.cnemc.cn/). There are nine national air quality monitoring sites located in Nanjing, including Maigao Bridge (MG), Caochangmen (CC), Shanxi Road (SX), Zhonghuamen (ZH), Ruijin Road (RJ), Xuanwu Lake (XW), Pukou (PK), Olympic Stadium (OS) and Xianlin University Town (XL). PM_2.5_ concentrations before publication have been gone through strict quality control by Ministry of Environmental Protection of the People’s Republic of China. Hourly concentrations of PM_2.5_, PM_10_, SO_2_, NO_2_, CO and O_3_ for these nine sites were collected from 1 March 2014 to 28 February 2017. The daily average concentrations of air pollutants were calculated only when there were more than 16 h of valid data. In this study, we defined spring as March to May, summer as June to August, fall as September to November, and winter as December to February. The quality assurance and control of state controlled monitoring data were reported in previous studies [34,35]. Meteorological data, including air temperature (T), relative humidity (RH), wind speed (WS) and atmospheric pressure (AP), were obtained from the China Meteorological Data Sharing Service System Administration (http://data.cma.cn/site/index.html).

### 2.2. Estimation of Premature Mortality Attributable to PM_2.5_

In this study, the causes of mortality were defined by the International Classification of Diseases 10 (ICD-10) code: IHD (I20-I25); stroke (I60-I67, I69.0, I69.1, I69.2, I69.3); lung cancer (C33, C34); COPD (J40-J44). The population data in Nanjing and the baseline mortality due to a specific disease of male and female population for each 5-year age group from 2014 to 2015 were obtained from the China Public Health and Family Planning Statistical Yearbook 2015 and 2016, respectively. Because the China Public Health and Family Planning Statistical Yearbook 2017 has not yet been published, the health burden estimation for 2016 was not conducted. Afterward, the relative risk (or risk ratio, *RR*) of several causes of premature mortality (COPD, LC, IHD and stroke) was estimated according to the IER functions [20]:

(1) $$RR(C)=\left\{ \begin{array}{ll} 1+\alpha (1-e^{-\gamma {\left(C-C_{0}\right)}^{\delta }}), & if\;C>C_{0}\\ 1, & else \end{array}\right.$$

where *C* is the ambient PM_2.5_ concentration, and *C*_0_ is the endpoint-specific theoretical minimum-risk concentration of PM_2.5_. 𝛼, 𝛾, and 𝛿 are parameters that determine the shape of C–R curves. A distribution of 1000 point estimates for *C*_0_, 𝛼, 𝛾, and 𝛿 parameters provided by the IER were utilized to calculate the mean *RR* and its 95% confidence intervals (CIs). The attributable fractions (*AF*) were than calculated as:

(2) AF=(RR−1)/RR

The premature (excess) mortality (Δ*Mort*) attributable to PM_2.5_ was calculated based on epidemiological cohort studies, as suggested by Anenberg et al. [29,36]:

(3) $$\Delta Mort=y_{0}\times AF\times Pop$$

where *y*_0_ is the baseline mortality rate (BMR) for a given population, *Pop* is the population within the age range of interest. The total deaths can be calculated as the sum of deaths caused by COPD, LC, IHD and stroke.

### 2.3. Calculation of YLL

YLL, an important part of disability adjusted life years (DALY), is a measure of disease burden considering life expectancy. Compared with the traditional measure of mortality, YLL gives more weight to deaths among younger people. We should pay more attention to the effects of PM_2.5_ on young people, who have a longer life expectancy than the elderly. At present, YLL is regarded as a more precise indicator to evaluate the burden of disease.

In this case, cause-specific YLL can be calculated by the DALY calculation template provided by the WHO, which calculated YLL from numbers of deaths using discounted and age-weighted life expectancies interpolated for the appropriate exact ages at death from the right-hand columns given in the standard life tables (http://www.who.int/healthinfo/global_burden_disease/tools_national/en/). The YLL template mainly contains the age group column, population column (yellow), deaths column (green), deaths per 1000 column, average ages at death column (blue), standard life expectancy column, YLL column and YLL per 1000 column. As the formula has been edited, we only need to replace the corresponding data to calculate the YLL, following the steps below: (1) Enter the population data in yellow cells below; (2) enter numbers of deaths for 5-year age groups in green cells below (or death rates in the next column and calculate numbers of deaths); (3) if necessary, modify average ages at death (blue column). The standard life expectancy was filled with the China life expectancy data provided by WHO. The total YLL was also the sum of the cause-specific YLL (COPD, LC, IHD, stroke). The YLL per 1000 people can be calculated by dividing the total YLL by the total population, then multiplying by 1000.

### 2.4. Evaluating the Effectiveness of PM_2.5_ Reduction

We also developed four reduction scenarios to calculate the potentially avoidable premature mortality by reducing ambient PM_2.5_ concentration in Nanjing to WHO IT1, IT2, IT3 and AQG standards. The annual PM_2.5_ concentrations of IT1, IT2, IT3 and AQG are 35, 25, 15 and 10 μg/m^3^, respectively. During this estimation, we assume that (1) the cause-specific mortality rates are independent of PM_2.5_ concentrations; (2) the population and age distribution also remain unchanged.

## 3. Results and Discussion

### 3.1. Characterization of PM_2.5_ Pollution in Nanjing

Annual average PM_2.5_ concentrations in 2014, 2015 and 2016 were 71 ± 35, 55 ± 33, 45 ± 29 μg/m^3^ (mean ± SD, calculated from daily values), respectively (Table 1). This downward trend on a yearly basis implied an improvement in the air quality of Nanjing after the implementation of China’s National Air Pollution Control Action Plan. However, the annual average concentration of PM_2.5_ in 2016 is still higher than the Chinese Ambient Air Quality Standards (CAAQS, GB3095-2012: 15 μg/m^3^ for Grade I and 35 μg/m^3^ for Grade II). The reduction and regulation of PM_2.5_ pollution is a long and arduous task. A remarkable seasonal variability was observed in Nanjing, where winter had the highest PM_2.5_ concentrations (74 ± 40 μg/m^3^) while summer had the lowest (45 ± 29 μg/m^3^). Such variations might be mainly caused by the enhanced anthropogenic emissions from fossil fuel combustion and biomass burning, the unfavorable meteorological conditions for pollution dispersion in winter (i.e., more frequent occurrences of stagnant weather and temperature inversion during the cold periods), as well as the pollutants transportation from Northern China under East Asian winter monsoon climate [7].

**Figure 1:**
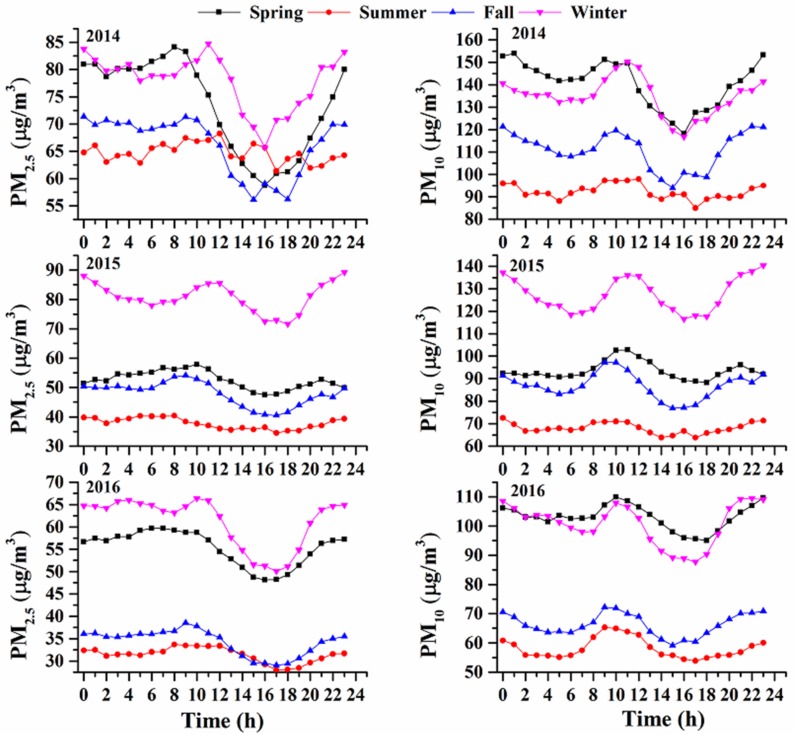
Diurnal variation of hourly PM_2.5_ and PM_10_ concentrations in 2014, 2015 and 2016.

Hourly data were used to examine diurnal variability in PM_2.5_ (Figure 1). Similarly, the diurnal variation in PM_2.5_ concentrations was more obvious in winter and spring than in fall and summer. The moderate PM_2.5_ peak appeared in the morning (i.e., 8:00 am to 11:00 am), in accordance with the morning rush hours, indicating that vehicle emissions make the main contribution to this pollution. From 12:00 to 16:00, the concentrations of PM_2.5_ decreased, and the lowest concentrations can be observed in the afternoon hours, mainly caused by the increasing boundary layer depth and the reducing of anthropogenic emissions in the afternoon. Later, PM_2.5_ concentrations peaked again due to the decreasing boundary layer heights and the evening rush hours. The changes in PM_10_ concentration showed a similar trend to those of PM_2.5_, but the peak of PM_10_ during rush hours was higher than PM_2.5_ peak, suggesting that vehicle emission made greater contributions to PM_10_ concentrations.

**Figure 2:**
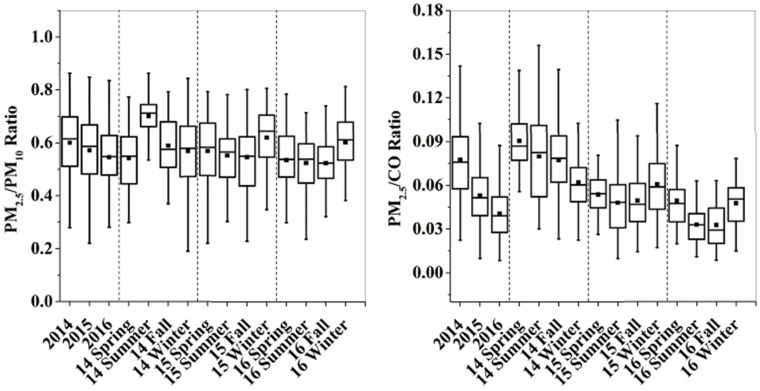
The statistical boxes for the ratios of PM_2.5_/PM_10_ and PM_2.5_/CO across different seasons in 3 years.

The ratios of PM_2.5_/PM_10_ and PM_2.5_/CO have been used to analyze the sources of PM_2.5_ pollution semi-quantitatively. Figure 2 shows the statistical boxes for the ratio of PM_2.5_/PM_10_ and PM_2.5_/CO across different seasons in 3 years. In general, the ratio of PM_2.5_ to PM_10_ slightly decreased from 2014 to 2016; in other words, the proportion of coarse particles increased, suggesting that the current PM_2.5_ control strategies (i.e., reduce fossil/non-fossil combustion derived VOCs and PM emissions) had a greater impact on PM_2.5_ pollution in Nanjing [7]. Moreover, this ratio was higher during the winter months of 2015 and 2016 in comparison to other seasons, suggesting the importance of combustion sources and the formation of fine particles of secondary origin in winter. The PM_2.5_/CO value is often used to discuss the secondary contribution to PM_2.5_ which excludes the influence of primary combustion emissions and meteorological factors [7]. The decreased ratios of PM_2.5_/CO from 2014 to 2016 showed that secondary contributions were better restrained under the implementation of PM_2.5_ control strategies in recent years. Comparable with PM_2.5_/PM_10_, the PM_2.5_/CO ratios in winter were slightly higher than that in other seasons, also demonstrating the contribution of secondary formation to PM_2.5_ in winter. Besides this, CO concentration is considered as an indicator of vehicle exhaust [37]. An obvious increasing trend can be found in CO concentration from 2014 to 2016, and winter often had a comparatively higher level of CO. When we combined our ratio data with the results of CO concentrations, we proposed that the relative increase of primary contribution to PM_2.5_ was partially caused by the increasing vehicle exhaust.

**Figure 3:**
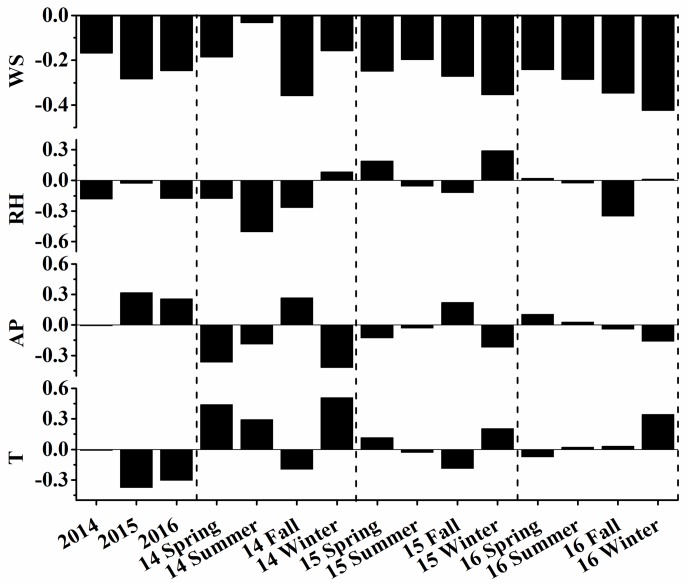
Correlations between PM_2.5_ and meteorological parameters: air temperature (T), air pressure (AP), relative humidity (RH) and wind speed (WS) across different seasons in three years.

Meteorological conditions have been demonstrated to play an important role in PM_2.5_ pollution [5,38,39,40]. Figure 3 shows the correlation coefficients between daily mass concentrations of PM_2.5_ and meteorological parameters across different seasons in three years. In general, the relationships between PM_2.5_ concentrations and meteorological parameters were complicated. PM_2.5_ concentrations were negatively correlated with wind speed in all cases, which indicates that strong horizontal dispersion played an important role in reducing PM_2.5_ pollution. Based on the annual statistics, PM_2.5_ concentrations exhibited negative correlations with relative humidity, and this was likely caused by the wet scavenging effects. However, in some seasons, positive correlations can be found, mainly because high relative humidity favors the formation of secondary aerosol from liquid phase reaction and moist condition usually accompanies with low boundary layer heights for particles accumulation [1,41,42]. PM_2.5_ concentrations were positively correlated with air pressure in the annual analysis, mainly because high air pressure restrained the upward movement of particles, leading to the accumulation of PM_2.5_ in the boundary layer. In general, temperature could affect the PM_2.5_ concentrations by affecting the dispersion or the formation of particles. The correlations between PM_2.5_ and temperature were different between annual analysis and seasonal analysis. In the annual analysis, there were negative correlations between PM_2.5_ and temperature due to the efficient vertical dispersion of pollutants under high temperatures. But in the seasonal analysis, positive correlations were found in some cases, likely caused by the formation of secondary particles via photochemical processes under higher air temperature conditions [1]. These variations indicated that the influence of temperature on PM_2.5_ is different.

### 3.2. Health Burdens Attributable to PM_2.5_

The relative risk (RR) for disease-specific mortality was estimated based on the IER model. The attributable fractions (AFs) of the premature mortality due to PM_2.5_ for these four diseases were then calculated using Equation (2) (Table 2). In 2014, the AFs (%) for COPD, LC, IHD, and stroke were 23% (95% CI 12–32%), 29% (95% CI 11–40%), 30% (95% CI 21–48%), and 46% (95% CI 17–57%), respectively. In 2015, with the decrease of PM_2.5_, the AFs had fallen to 20% (95% CI 10–29%), 25% (95% CI 8–35%), 28% (95% CI 19–44%), and 44% (95% CI 15–55%). Consistent with previous studies, in the current environment, AF was the largest for stroke, followed by IHD, LC and COPD [23,24,27,28]. There is still significant scope for a decline of AFs if the government continues to improve ambient air quality to meet the WHO IT-1, IT2, IT3 and AQG standards. When PM_2.5_ was <15 μg/m^3^ (IT-3), IHD instead of stroke became the major cause of death.

**Figure 4:**
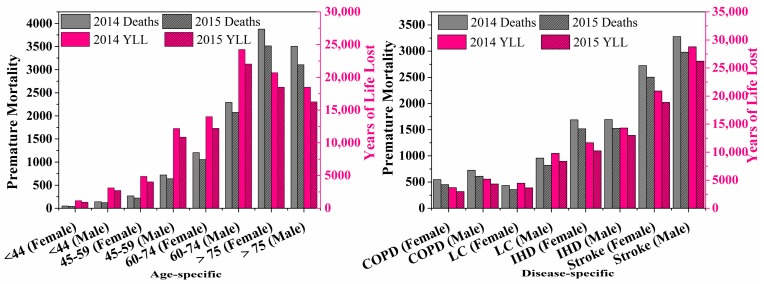
Premature mortality and years of life lost attributable to PM_2.5_ in Nanjing.

Based on the disease-specific mortality and the population data in Nanjing, we estimated the mortality burden attributable to current ambient PM_2.5_ exposure in the year 2014 and 2015. Figure 4 shows the premature mortality caused by IHD, stroke, COPD and LC for males and females. The total deaths from IHD, stroke, COPD and LC in Nanjing attributable to PM_2.5_ were 12,055 and 10,771 in 2014 and 2015. The disease-specific deaths for IHD, stroke, COPD and LC in 2014 were 3382, 6002, 1276 and 1395, respectively. The deaths caused by PM_2.5_ decreased to 3043, 5485, 1064 and 1179, respectively, due to the reduction of AFs in 2015. Stroke was the major cause of death, followed by IHD, similar to the results from a national estimation [22]. The occurrence of these diseases suggested that PM_2.5_ not only injure the respiratory system but also significantly affect the cerebrovascular system. Moreover, there existed significant gender differences in the mortality burden: disease deaths in males were generally higher than that in females. It should be noted that LC has a long latent period [43], and that LC could be compounded by smoking; the health burden for LC in males deserves more attention. The differences mainly resulted from the gender-specific baseline mortality rate. Figure 4 shows the numbers of premature deaths in different age categories. The elderly (>60) accounted for a substantial proportion of total deaths, especially as people older than 75, while the youngers (<44) contributed little to the deaths. This is mainly caused by the differences in baseline mortality rate among the different age groups, as the older people are more sensitive to PM_2.5_ exposure.

Long-term exposure to PM_2.5_ contributed to 98,802 and 87,647 years of life lost in 2014 and 2015, respectively. Relative contributions of COPD, LC, IHD and stroke to YLL are shown in Figure 4. In general, additional YLL attributable to PM_2.5_ decreased from 2014 to 2015, and the lost years were also higher in males than in females, mainly because of a higher all-cause mortality rate in males. Similar to the premature death, the contribution of stroke to YLL was the highest, followed by IHD, suggesting that cardiovascular disease (stroke and IHD) instead of respiratory diseases (COPD and LC) accounted for the majority of deaths and YLL attributable to ambient PM_2.5_ air pollution. YLL per 1000 person for COPD, LC, IHD and stroke were 1.08, 1.73, 3.16 and 6.05 years in 2014, and 0.89, 1.46, 2.82 and 5.48 years in 2015, respectively. As expected, YLL per 1000 person was higher in males than in females, and the contributions of cardiovascular disease were higher than that of respiratory diseases. Figure 4 shows the contributions of deaths in different ages to YLL. Differently to the premature mortality, 60–74 gerontism contributed more to YLL than the gerontism (>75) did. YLL in Nanjing (estimated in this study) was about 1065 years per 100,000, higher than the numbers of developed countries, such as USA (337.1 years per 100,000), Brazil (573.7 years per 100,000), Japan (261.7 years per 100,000) in 2015 estimated by Cohen et al. [28]. These results implied that health burden associated with PM_2.5_ in Nanjing was serious, and more rigorous regulations should be taken in Nanjing.

**Figure 5:**
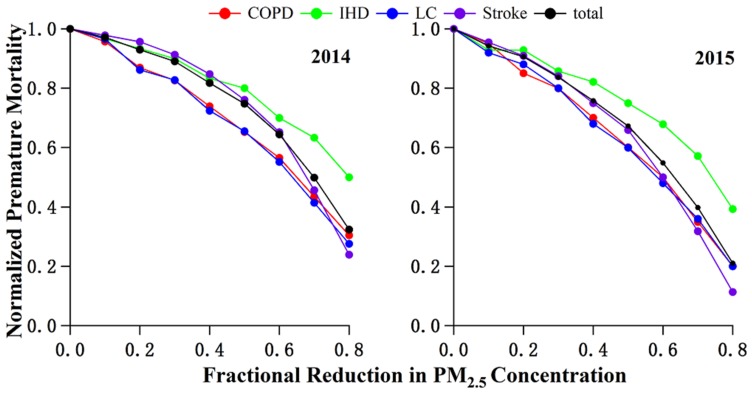
Normalized premature mortality as a function of fractional reduction in PM_2.5_ concentrations.

Figure 5 estimates the avoidable premature mortality when PM_2.5_ concentrations are reduced to different levels. A 50% reduction in PM_2.5_ concentrations in 2014 would lead to 35%, 20%, 35% and 24% reductions in premature mortality for COPD, IHD, LC and stroke, respectively. The corresponding reductions in premature mortality were 40%, 25%, 40% and 34% from the 2015 level. To achieve a 50% reduction in COPD, IHD, LC and stroke-caused excess mortality, PM_2.5_ concentrations need to be reduced by 64%, 80%, 64% and 68% relative to the 2014 level, and 60%, 74%, 58% and 60% relative to the 2015 level, respectively. The benefits of reducing PM_2.5_ for COPD and LC were higher than those for stroke and IHD. Besides this, the excess mortality as a whole decreased slowly in the beginning, but the marginal benefit grew faster with a progressive reduction in PM_2.5_ concentrations. We can conclude that higher benefits could be obtained by a proportionate reduction in PM_2.5_ when initial PM_2.5_ concentration was lower, and these results were consistent with earlier research by Hu et al. and Joshua et al. [22,23].

**Figure 6:**
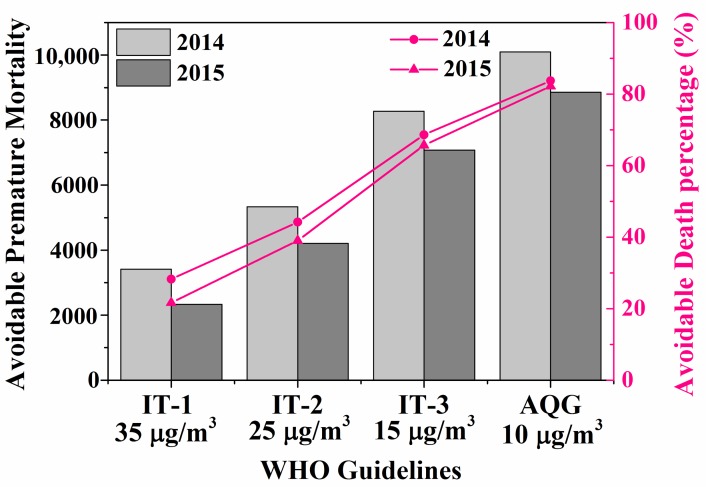
Potentially avoidable premature mortality and relative percentage if the PM_2.5_ concentrations were reduced to the World Health Organization (WHO) three interim targets (ITs) and air quality guidelines (AQG).

Figure 6 describes the potential mortality benefits when PM_2.5_ concentrations in Nanjing were reduced to different levels. The PM_2.5_-related premature mortality in Nanjing would be reduced by 28% from 12,055 to 8646 when the concentrations of PM_2.5_ in 2014 meet the current CAAQS grade II standard (or WHO IT1, 35 μg/m^3^). Under stricter standards, the reductions in premature deaths were 44%, 69%, and 84% when meeting the standard of 25 μg/m^3^ (WHO IT2), 15 μg/m^3^ (WHO IT3), and 10 μg/m^3^ (AQG), respectively. The avoidable premature deaths in 2015 were slightly lower than that of 2014, and males were still at higher health risk than females. Even if the PM_2.5_ concentration meets the Chinese National Grade II standard (35 μg/m^3^) in the next few years, it could still contribute to more than eight thousand total deaths in Nanjing, with relative high premature mortality. Satisfactory decline could be found only when meeting stricter standards. Considering the huge health benefits, more rigorous control measures should be taken in Nanjing to improve the air quality.

Estimation of health burden in our study was based on the most reliable notification data, including PM_2.5_ concentrations and population data. There were still some limitations and uncertainties, however, like all model studies. First, a comparison has been made between the RRs observed in China and those calculated by the IER model. Although the IER model yielded reasonable results in China [21,22,26,27], the concentration–response function obtained by epidemiology studies conducted at a city level should be developed in the future. Second, the toxicity of PM_2.5_ varied according to its chemical composition. Risk assessment would be better to use the chemical composition data, but these data were not available. More studies are needed to investigate the long-term health effects related to PM_2.5_ chemical component. Third, changes in the model parameters (e.g., baseline mortality rate, population and age distribution) were neglected in benefits analyses of PM_2.5_ reduction. Despite these limitations, our study highlights the necessity to improve air quality to effectively reduce mortality.

## 4. Conclusions

This paper analyzed the temporal characteristics of PM_2.5_ in Nanjing from March 2014 to February 2017, as well as their relationships with meteorological parameters. Then, the IER model was applied to assess the premature mortality and YLL due to PM_2.5_. Furthermore, the mortality benefits were estimated under different scenarios of meeting the standards of WHO IT1, IT2, IT3 and AQG.

Annual average PM_2.5_ concentrations in 2014, 2015 and 2016 were 71 ± 35, 55 ± 33 and 45 ± 29 μg/m^3^ (mean ± SD, calculated from daily values), respectively, which showed a continuous decrease on a year to year basis. A remarkable seasonal variation (maximum in winter and minimum in summer) in PM_2.5_ concentrations can be found during our study periods. Diurnal variations showed that the concentrations of PM_2.5_ were mainly influenced by anthropogenic emissions, diffusion conditions, and secondary formations. The ratios of particles to gaseous pollutants suggested that secondary contributions had been effectively regulated, while the relative contributions of vehicle exhaust increased in recent years. There were negative relationships between PM_2.5_ concentrations and wind speed, but the correlations between PM_2.5_ and other meteorological factors varied as per annual analysis and seasonal analysis, and their combined effects need to be further considered.

The total premature mortality in Nanjing caused by PM_2.5_ were 12,055 and 10,771 in 2014 and 2015, respectively. Stroke was the leading cause of death, followed by IHD, LC and COPD. Generally, disease deaths in males and the elderly were higher than that in females and youngsters, indicating that the former two are more sensitive to PM_2.5_ pollution. Long-term exposure to PM_2.5_ resulted in 98,802 and 87,647 years of life lost in 2014 and 2015, respectively. To achieve a 50% reduction in COPD, IHD, LC and stroke-caused excess mortality, corresponding reductions of PM_2.5_ concentrations by 64%, 80%, 64% and 68% relative to the 2014 level, and 60%, 74%, 58% and 60% relative to the 2015 level, respectively, are necessary. When the concentrations of PM_2.5_ in 2014 meet the current CAAQS grade II standard of 35 μg/m^3^, the related premature mortality in Nanjing would be reduced by 28%, and higher health benefits could be achieved if Nanjing adopted more stringent guidelines. According to our results, the health burdens associated with PM_2.5_ in Nanjing were higher than that in developed countries, and more rigorous regulations should be taken in Nanjing to ameliorate existing pollution.

## Figures and Tables

**Table 1:** Annual and seasonal average fine particulate matter (PM_2.5_) concentrations during the study period (μg/m^3^, mean ± SD, calculated from daily values).

	Annual Mean	Spring	Summer	Fall	Winter
2014	71 ± 35	74 ± 29	65 ± 34	67 ± 34	78 ± 42
2015	55 ± 33	53 ± 21	38 ± 21	47 ± 28	81 ± 42
2016	45 ± 29	55 ± 32	31 ± 16	34 ± 19	61 ± 32
Average	57 ± 34	60 ± 29	45 ± 29	49 ± 31	74 ± 40

**Table 2:** The attributable fractions due to PM_2.5_ for chronic obstructive pulmonary disease (COPD), ischemic heart disease (IHD), lung cancer (LC) and stroke under target scenarios.

	PM_2.5_ (μg/m^3^)	Attributable Fractions (AFs) (%)			
		COPD	IHD	LC	Stroke
2014	71	23	30	29	46
2015	55	20	28	25	44
2016	45	18	26	22	41
IT-1	35	15	23	18	34
IT-2	25	11	20	14	25
IT-3	15	7	15	8	11
AQG	10	3	10	4	4
